# Estimation of mobility and population in Spain during different phases of the COVID-19 pandemic from mobile phone data

**DOI:** 10.1038/s41598-023-36108-1

**Published:** 2023-06-02

**Authors:** Joaquín Osorio Arjona, Julia de las Obras-Loscertales Sampériz

**Affiliations:** 1 Department of Human Geography, University of Sevilla, 41004 Seville, Spain; 2 Consejo Superior de Investigaciones Científicas, Centro de Ciencias Humanas y Sociales (CCHS-CSIC), 28037 Madrid, Spain

**Keywords:** Microbiology, Information technology

## Abstract

This work aims to find out the effectiveness of sources based on Big Data like mobile phone records to analyze mobility flows and changes in the population of Spain in different scenarios during the period of the pandemic caused by the COVID-19 virus. To this end, we have used mobile phone data provided by the National Institute of Statistics from four days corresponding to different phases of the pandemic. Origin–Destination matrices and population estimation calculations at the spatial level of population cells have been elaborated. The results show different patterns that correspond to the phenomena that have occurred, as the decrease of the population during the periods associated with the confinement measures. The consistency of findings with the reality and the generally good correlation with the population census data indicate that mobile phone records are a useful source of data for the elaboration of demographic and mobility studies during pandemics.

## Introduction

The COVID-19 pandemic has radically changed people's daily lives. Within weeks of the start of the pandemic, discussions about the use of geolocated data and visualizations of global mobility flows became a recurring theme in the media, and government agencies and companies began using geolocated data to track the spread of the disease^1,2^. In this situation, studying population and mobility patterns is of great value to be able to see which areas are particularly affected by COVID-19 in different periods of the disease, and to be able to take appropriate health measures. It is important to visualize local differences to study the transmission of this type of infectious disease since it must be considered that contact patterns and population size vary in the different regions of a country^3^. However, working with large data samples of infected and uninfected individuals usually takes months to process, and the results obtained hardly reach the public, who are usually unable to follow the work of the health community^4^. In addition, traditional sources for capturing disease-related data do not capture cases in which sick people are unable to visit health centers^3^.

Compared to classical survey-based methodologies, the advantages of using geo-localized Big Data are the availability of large data samples with high spatial and temporal detail, in a short time and at low cost^2,5,6^. These data allow to monitor in near-real time the activity of the population^7^. Mobile phone call data records (CDR) are one of the most used Big Data sources in large-scale projects, due to their larger sample size and higher temporal resolution of their data, with a record produced in an interval of a few seconds that allows the study of more complex mobility patterns^8–10^.

In studies related to the COVID-19 pandemic, CDRs are primarily used to reveal patterns and trends in demographics and mobility over space and time, to examine the role of human mobility with the spread of the virus, and to perform simulations and predictions of disease transmission^11^. The combination of GIS and Big Data play an important role in several aspects, such as rapid aggregation of different sources, visualization and mapping of epidemiological information, spatial tracking of confirmed cases, prediction of transmission at the regional level, and spatial segmentation of the effects of the epidemic for the design of prevention policies at regional levels^12^. Ultimately, mobile phone data provide the means to study virus transmission, understand daily changes in demographics and mobility, and visualize recovery processes^2^. The usefulness of this data is constantly being demonstrated, and mobile phone operators are increasingly receptive to providing access to this type of data for the development of projects^13^.

This work therefore seeks to use mobile phone data as an alternative, fast, and high-volume data source to analyze the changes in demographic patterns in Spain during different phases of the pandemic caused by the COVID-19 virus. The research question consists of finding out the effectiveness of these new sources based on Big Data in obtaining and visualizing behaviors associated with each of the periods of the disease. It is based on the starting hypothesis that the reduction in mobility and the increase in mortality have led to a decrease in the number of mobile phone data in the phases with greater association to states of alarm and restrictions, and subsequently, to a population decline.

In comparison with other works, this research analyzes and maps the results obtained during four differentiated moments that have occurred within a period of 15 months, using a spatial scale that allows the visualization of mobility and population patterns in different areas of the territory. Also, this work validates the performed analysis using determination coefficients and residue mapping. The following paper is divided into five sections. Following this introduction, “Literature review” section will provide a state of the art of the issue, “Study area” section will present the study area and the data used, and the methodology employed in the research will be reviewed. Finally, in “Results” section, the results obtained will be analyzed and a series of conclusions will be drawn in “Conclusions” section.

## Literature review

Geolocated Big Data, specifically CDR data, is a tool that has been employed since the past decade in various research on the spread of infectious viral diseases in space, mainly in developing countries in Africa, Latin America, and Southeast Asia) such as influenza^14^, malaria^15^, dengue^16,17^, cholera^18^ or ebola^19^.

The high relevance of the spread of COVID-19 in space and time has led to the publication of a relevant number of investigations that have employed CDR data for the visualization of mobility patterns during the pandemic. In contrast to previous research that has dealt with other infectious diseases in developing countries, a significant number of COVID-19-related studies have focused on the United States or European countries as study areas, with the main result being the decrease in population movements after the onset of the pandemic. Thus, Kang et al. have designed Origin–Destination (OD) matrices at different U.S. spatial scales (census tract, county and state) and temporal scales (week and day scale) and validated the results with the mobility flows of the American Community Survey^20^. Lee et al. have used more than 100 million mobile phone records to establish location clusters at possible home and work locations in the United States, resulting in heterogeneity in mobility flows across income density groups^21^.

In Europe, Jeffrey et al. have employed both mobile phone data from the *O2* company and data from the *Facebook* mobile phone application, finding that both data sources give similar results, albeit with differences with respect to identifying the cities with the highest number of registrations^22^. Santamaria et al. used the data requested by the European Commission from the phone companies to analyze the impact of the mobility restriction measures established by the different European countries. The results obtained determined that in most cases, confinement measures explain more than 90% of the mobility patterns obtained^23^. Willberg et al. studied the correlation between the number of records obtained on weekdays and weekends and the correlation between the weekday population and the rate of second homes per municipality. The results showed a 10% decrease in mobility in the country and a migration from the most populated cities to second home areas^24^. Szocska et al. obtained similar results using Hungary as the study area. They also designed a mobility index and a stay-at-home index, obtaining a pronounced decrease in mobility on weekends^25^.

In other countries around the world, Peixoto et al. compared CDR from the Brazilian states of São Paulo and Rio de Janeiro in March of the years 2019 and 2020. In addition to the clear decline in mobility, they found that most of the movements occurred in the dormitory cities surrounding the main cities of the metropolitan areas^26^. Lawal and Nwegbu used both mobile phone data and *Google* mobility report data to examine in Nigeria mobility trends in different locations categorized as stores, parks or pharmacies using correlation analysis and multiple correspondence analysis. They observed a sharp decline in mobility in more than half of the country, in addition to a prolonged increase in mobility over time in supermarkets and entertainment venues^27^. Y. Zhou et al. compared phone records from the first three months of 2019 with COVID-19 case reports published in the first quarter of 2020 and designed a transmission model to predict the spread of the virus in the city of Shenzhen^28^. Nagata et al. analyzed the daily mobility change in work, nightlife, and residential locations in the three major cities of Japan (Tokyo, Osaka, and Nagoya). They found that the main change in population mobility patterns occurred during the nighttime period^29^.

It should be noted that the time period of these papers is limited to the first few months of the pandemic (generally March to May 2020). Few papers are known to have used a broader time scale. In this regard, Kim and Kwan have used county-level US mobile phone data from March 1 to September 30, 2020, and enriched them with other socioeconomic variables. They found a smaller reduction in mobility in the population with low-income levels and a gradual increase in overall mobility once the initial alarm state had passed^30^. Kephart et al. used aggregated phone data from cities with more than 100,000 inhabitants in Argentina, Brazil, Colombia, Guatemala and Mexico from March 2 to August 29, 2020. They estimated that these data allowed them to cover about 5% of the population in each country. They also used reports of confirmed COVID-19 cases provided by the governments of the study cities, in addition to other socioeconomic data such as educational level or population density^31^.

Regarding Spain as the study area, Pérez-Arnal et al. have used mobile phone data from March to June 2020 provided by the company *Apple*, in addition to *Facebook* app data and *Google* mobility reports. The results consist of strong mobility changes on weekends, mobility recovery mainly on weekends after the first phase of the pandemic, and important changes on weekdays in urban regions with high population density, being these cities the areas with the most persistent mobility reduction over time^32^. Mazzoli et al. (2020) used 13 million mobile phone records from *Kido Dynamics SA* for the first phase of the pandemic. These data were aggregated by province of origin and combined with mobility data from epidemiological reports of the *Escovid19data* project. The results showed an increase in COVID-19 incidence and mortality in the weekend following the establishment of containment measures, and in mobility flows with the province of Madrid as both origin and destination^33^. Romanillos et al. used combined mobile phone data with land use data from the cadastre. From them, the population distribution was compared according to cadastral land use and time of day, Ordinary Least Squares (OLS) analysis were used to compare the population distribution at each time of the week, and a Spatial Multiple Regression Analysis to analyze the relationship between population presence and land use. The results obtained included an increase in population in activity areas and a decrease in activity in residential areas throughout the day during a period of normal mobility while during the pandemic the activity areas did not attract population during the day while activity in residential areas did not decrease^24^.

The originality of this research lies first in the temporal scope employed. While all work on record to date has employed mobile phone data up to September 2020 at the latest, concentrating on the first alarm state of the pandemic, this research employs phone data to study mobility and population distribution in later periods (up to June 2021), including new phases of the pandemic such as the second alarm state caused by the increase in COVID-19 transmission in autumn 2020, or the transition to a stage of "new normal" caused by the availability of vaccines. In terms of spatial scale, the present work establishes a series of cells with a given number of inhabitants set between specific population thresholds, so that changes in mobility and population in the study country can be mapped as homogeneously as possible. Another novelty is the inclusion of a methodology for weighting a population sample based on mobile phone data in order to estimate a specific number of inhabitants on a particular day. In addition, this work includes a method for validating mobile phone data with respect to official population data by mapping residuals obtained by OLS analysis, a method observed only in Romanillos et al.^24^ at the urban scale and using other variables instead of official population data.

## Study area

**Figure 1:**
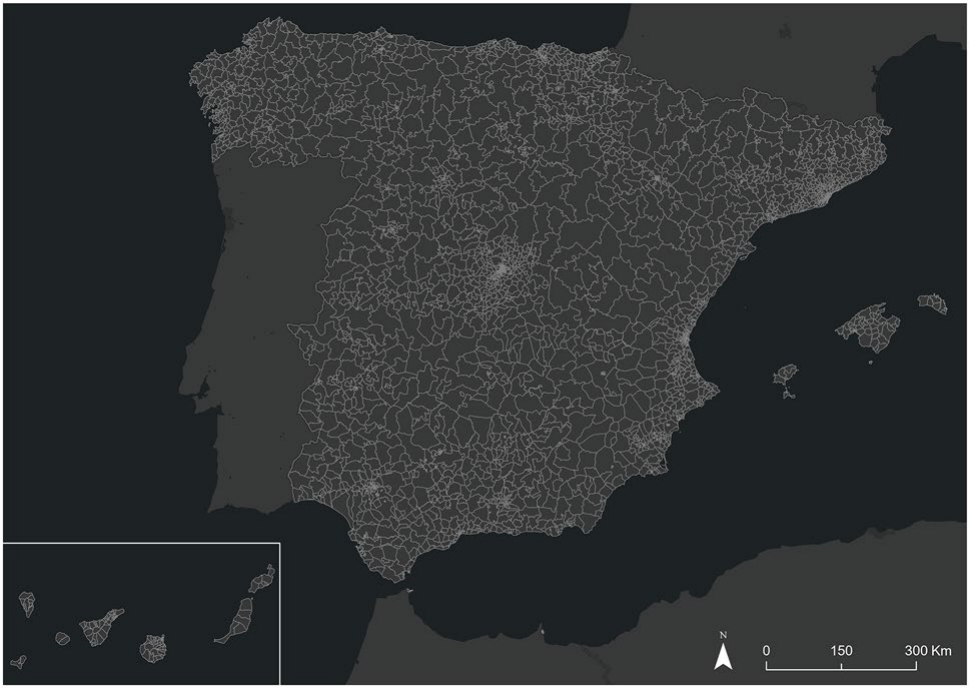
Population cells for Spain designed by the INE. *Source*: Own elaboration based on INE data from the *Estadistica Experimental* portal. The program used to create the figure is *ArcGIS Pro 3.0.3* GIS software. The figure displays the 3214 population cells designed by INE. These cells have a population referring to January 1, 2020, and have been designed to have a homogeneous population that exceeds the threshold of 5,000 registered inhabitants and does not exceed a threshold of 50,000 inhabitants.

Spain is a suitable country for studies related to the effects of COVID-19 due to its large and rapid pandemic peak and the implementation of several confinement and mobility restriction policies throughout the alarm state^32^. We have worked at a spatial scale of 3214 population cells designed by INE. These cells have a population referring to January 1, 2020, and have been designed to have a homogeneous population that exceeds the threshold of 5000 registered inhabitants and does not exceed a threshold of 50,000 inhabitants. As a result, Spanish cities are divided into several population cells (large cities such as Madrid and Barcelona have hundreds of cells), while sparsely populated municipalities in the interior of the peninsula are aggregated into a single cell. This methodology also alleviates the limitation of spatial dependence of mobile cell records on phone antennas (Fig. 1).

The time period for this research corresponds to four working days (Wednesdays) that correspond to four different periods of the pandemic caused by the COVID-19 virus through which the Spanish population has passed:

- April 15, 2020: day belonging to the first state of alarm period between March 15 and June 21, 2020, when the population of Spain was under quarantine and confinement measures, including travel restrictions and store closures (Royal Decree 463/2020, of March 14, declaring the state of alarm for the management of the health crisis situation caused by COVID-19). There are 3,578,378 records published on that day.
- August 19, 2020: day in which a considerable increase of mobility flows for summer vacation takes place annually. The lockdown at national level had already ended and a new stage called new normality had been entered, in which, although there were no longer mobility restrictions, preventive measures such as teleworking continued to be promoted. A total of 38,163,485 phone records were obtained on that day.
- December 16, 2020: day located in the second state of alarm, a period that lasted from October 25, 2020, to May 9, 2021. On these dates, mobility restriction measures were established based on the prohibition to leave the autonomous community of residence, causing different population and mobility patterns in the Spanish territory (Royal Decree 926/2020, of October 25, declaring the state of alarm to contain the spread of infections caused by SARS-CoV-2).
- June 9, 2021: period after the second state of alarm, with the definitive lift of mobility restrictions. This period is highlighted by the vaccination campaign against COVID-19 in Spain. At the beginning of June, the population over 50 years of age has already had access to the vaccine, which was beginning to be supplied to the rest of the age groups (COVID-19 vaccination strategy promoted by the Governement of Spain). By that day, there were 39,819,645 phone records.

## Data and methodology

For this work we have used mobile phone data collected and made available on its website by the INE from March 16, 2020, to December 29, 2021. These data come from the three main phone operators of Spain: *Movistar*, *Vodafone*, and *Orange*. Data from foreign numbered phones operating in roaming have been excluded. As of June 23, 2020, only records published on Wednesdays and Sundays are available. In particular, the tables with daily mobility data have been used. Each record corresponds to an OD (Origin–Destination) trip. OD matrices are especially useful to assess the mobility of the population during different epidemiological phases. Each OD trip register has a date, an identifier of the origin population cell, an identifier of the destination population cell, and a given number of mobile phones. These tables already have a first filter in which trip flows with less than 15 people detected have been omitted. The data downloaded from the INE web page are already separated by the day on which they were created, so we have worked directly with the tables of the four selected working days for the analysis of mapping of results.

The first step of the methodology has been to incorporate in the *ArcGIS Pro* GIS desktop a layer with the geometry, spatial and demographic information of the cells provided by the INE. Then, the centroids of the 3214 population cells have been calculated and an OD matrix layer (with a total of 10,329,796 relations) has been designed. Next, a *Python* script was developed to process, clean, enrich and correct the data of the four downloaded tables, eliminating the records whose destination cell has a value of *OTHER* (other destinations not corresponding with the population cells). Once the record tables were processed, they were incorporated into the GIS and joined to the OD matrix layer created in order to obtain four OD matrices layers. With these layers, it is possible to map both external and internal mobility flows during the four studied days.

To calculate the number of mobile phones in each population cell, the records of each table were summarized both by cell of origin and destination. By anonymizing and aggregating mobile phone data, not only do they not reveal information about individuals, but epidemiologically relevant estimates of population mobility are obtained^10^. As a result, a table of records per spatial cell has been created for each day, in which each record counts the number of mobile phones entering and leaving the population cell on the day of registration. Then, the estimated number of mobile phones $p^{m}$ of every population cell during each day was calculated using the following formula:

$$p^{m} = p^{s} + (p^{d} - p^{o} )$$

where $p^{s}$ is the stock of the number of mobile phones detected in the day (number of mobile phones that moved internally within the population cell), and $(p^{d} - p^{o} )$ is the mobile population balance happening during the day, composed by a number of mobile phones $p^{d}$ entering in the spatial cell, and a number of mobile phones $p^{o}$ leaving the cell.

Once the number of mobile phones in each population cell was calculated, the total population $p^{e}$ was estimated using the next formula:

$$p^{e} = \frac{{\left( {p^{m} *100} \right)}}{{w^{l} }}$$

where the number of mobile phones $p^{m}$ of each cell was weightened with a correction factor value $w^{l}$. This value is based on the percentage of the population who has used a mobile phone from the three main companies that support the data downloaded from the INE at the provincial level $l$. This factor value varies according to the province, so every population cell located in a determined province will use the same percentage value. These percentages were obtained from the National Commission of Markets and Competition (CNMC) data website.

The reason for this weighting methodology lies in the assumption that the bulk of the population using mobile phone data is in the 15–65 age range, so the INE mobile phone data do not have records based on the child or elderly population. In this way, the aim is to alleviate the problems of bias that mobile phone data usually have.

Once the population in each cell for each day of study has been estimated, the percentage increase in population $p^{g}$ has been mapped using the following formula:

$$p^{g} = \left( {\frac{{\left( {p^{e} - p^{c} } \right)}}{{p^{c} }}} \right)*100$$

where $p^{e}$ is the previously estimated population and $p^{c}$ corresponds with the population data of the census from the year 2020 supplied by INE.

The census population data for each cell was used to validate the results obtained by calculating R^2^ coefficients of determination with respect to the estimated population data $p^{m}$ based on unweighted mobile phone data. An OLS analysis was used to map the overestimation or underestimation of the estimated $p^{e}$ weightened values with respect to the censal population. Lastly, a Moran I spatial autocorrelation analysis was performed to analyse if the residual values obtained in the OLS analysis are spatially related.

## Results

**Figure 2:**
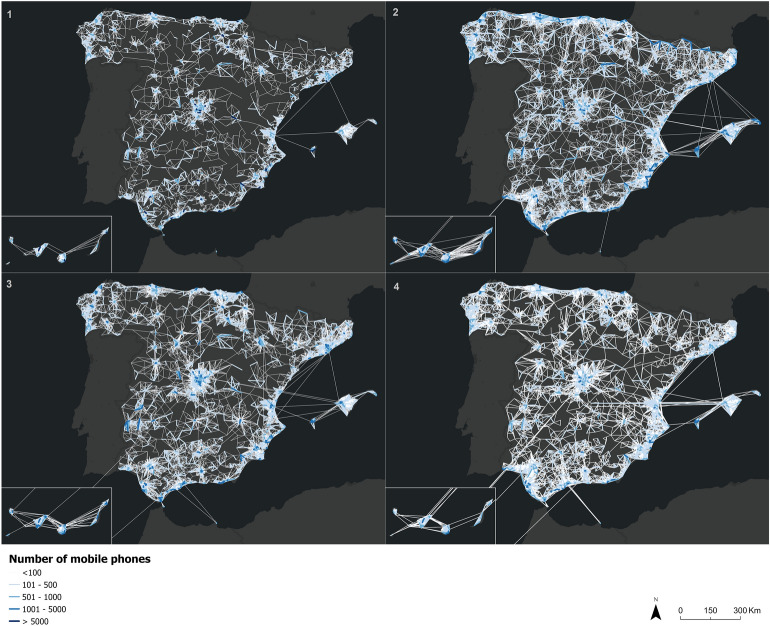
Number of mobile phones that have moved between different population cells on April 15, 2020 (1), August 19, 2020 (2), December 16, 2020 (3), and June 9, 2021 (4). *Source*: Own elaboration based on INE phone data from the *Estadistica Experimental* portal. The program used to create the figure is *ArcGIS Pro 3.0.3* GIS software. The figure shows an Origin–Destination matrix that indicates the number of mobile phones that have moved from a population cell to another one for the four mentioned days.

The first results allow us to identify the periods of the pandemic affected by quarantines and mobility restriction measures as the dates with the lowest number of cell phones recorded in the mobility flows. Thus, a reduced number of flows can be observed in April 2020, highlighting only trips in large metropolitan areas. On the other hand, with the end of the quarantine and the arrival of the summer period, an increase in trips can be observed in the archipelagos, the Cantabrian Sea coast and the Andalusian coasts. With the second alarm state in autumn 2020, a reduction in the number of trips is again seen, with mobility being concentrated in the large Spanish metropolitan areas. But contrary to the mandatory quarantine period of the first alarm state, a greater number and intensity of flows can be seen. This result is an indicator that, althought new mobility restrictions were activated, these restrictions were laxed than the measures in force during the first alarm state. In addition, the number of workers telecommuting decreased while there was an increase of the number of workers who physically traveled to their workplace. Finally, during the period after the second state of alert, in June 2021, a return to normal mobility behaviors can be visualized, highlighting an increase of the number of flows between cells (the Guadalquivir and Ebro valleys, the Galician Atlantic coast, and the Valencian Community stand out in comparison with previous periods) (Fig. 2).

**Figure 3:**
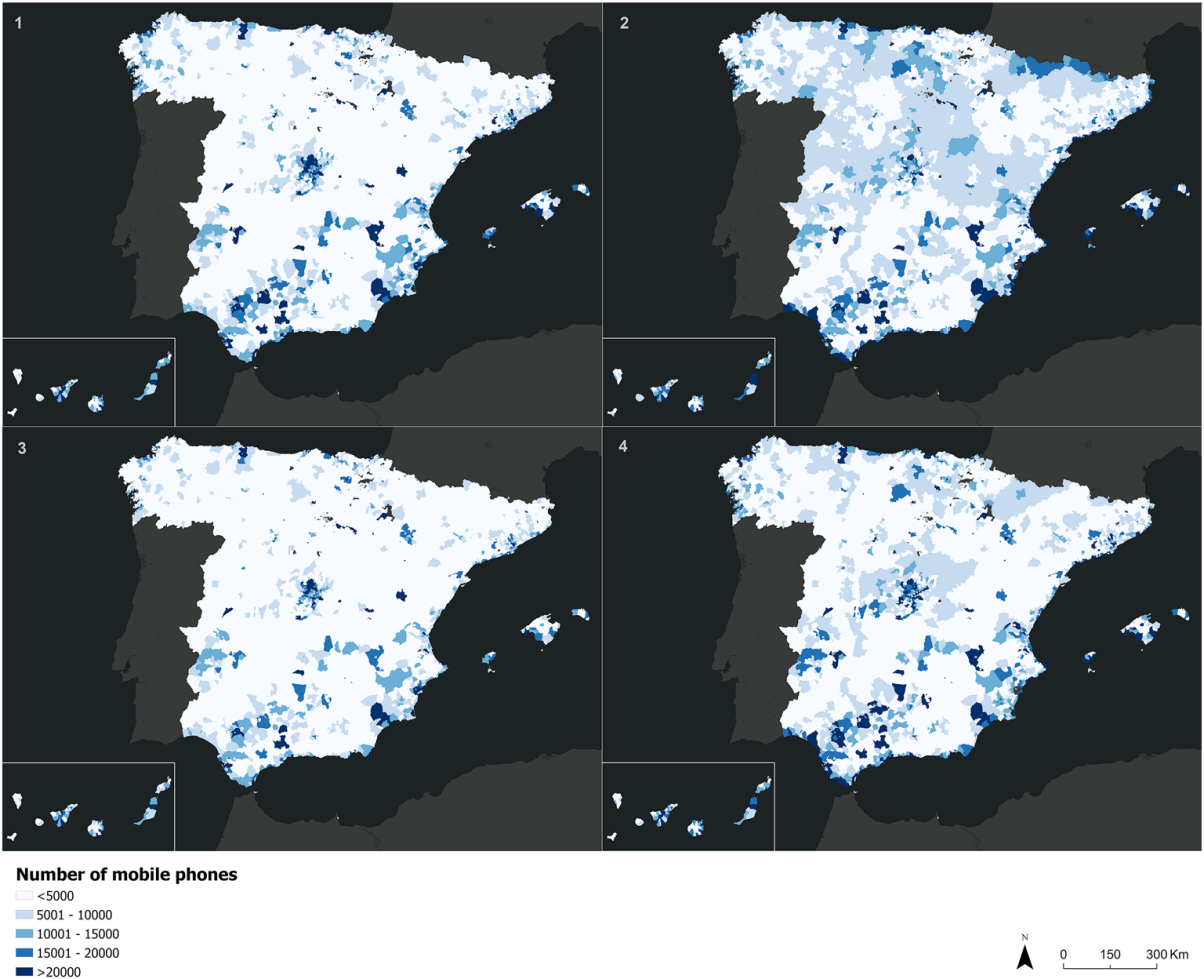
Mobile phone stock (number of mobile phones internally moved in population cells) on April 15, 2020 (1), August 19, 2020 (2), December 16, 2020 (3), and June 9, 2021 (4). *Source*: Own elaboration based on INE phone data from the *Estadistica Experimental* portal. The program used to create the figure is *ArcGIS Pro 3.0.3* GIS software. The figure is a choropleth map that indicates the number of mobile phones that have moved internally in a population cell for the four mentioned days.

If we look at the internal trips or stock of mobile phones during the two states of alarm this number was considerably lower, with a significant number of mobile phones moving mainly in the large metropolitan areas of Spain. This result indicates that phone users traveled mainly to local equipments in their home cell (shops, pharmacies, hospitals, etc.), while they were telecommuting, reducing the number of trips to workplaces located in other cells. On the other hand, during the dates without restrictions (mainly in the period after the first state of alarm), the number of phones increases significantly in other areas such as the Guadalquivir Valley, the Galician coast, or the peninsular interior (during August 2020). We can associate these places as reception places for Spanish tourists, but also with second residence zones (particularly in the case of the peninsular interior) (Fig. 3).

**Figure 4:**
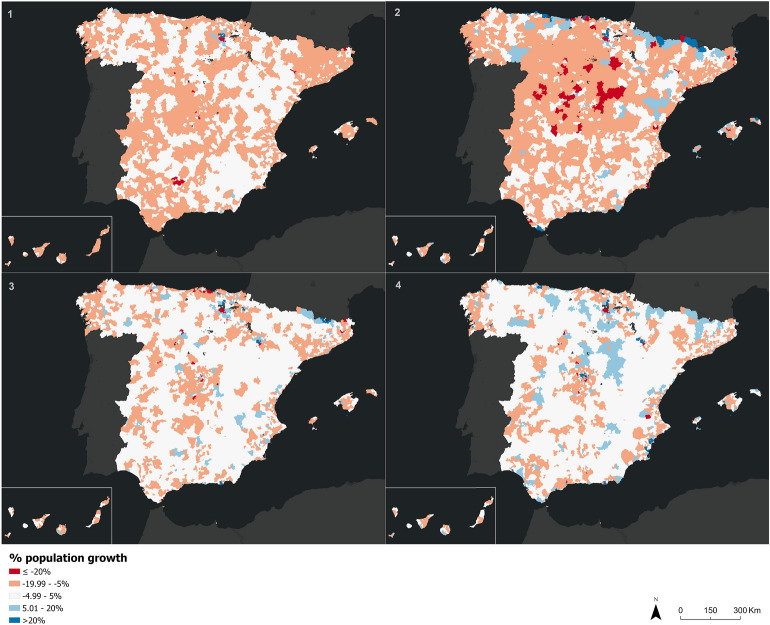
Percentage of estimated population growth over INE population by cell on April 15, 2020 (1), August 19, 2020 (2), December 16, 2020 (3), and June 9, 2021 (4). *Source*: Own elaboration based on INE phone data from the *Estadistica Experimental* portal. The program used to create the figure is *ArcGIS Pro 3.0.3* GIS software. The figure is a choropleth map that shows the growth of the estimated number of inhabitants in a population cell with respect to the censal population for the four mentioned days.

Analysing the demographic changes observed from the percentage of estimated population growth over the official populaton according to INE data, it’s noteworthy how the percentage of population has been reduced in a generalized way throughout the country during the first state of alarm. Moreover, in the months following this first phase, this percentage has been significantly negative in the mountain range of the Central System. These results indicate a generalized reduction in the number of inhabitants throughout the country and concentrated in the interior of the peninsula, which can be associated with the high mortality suffered by Spain in this first phase of the pandemic. The population reduction is softened throughout the country during the second state of alarm, with many inland cells experiencing a situation of zero growth. In August 2021, the population growth in Spain recovered and we see a bigger number of cells with a positive increase in the number of inhabitants. In this new phase, the cells with a decrease in population are concentrated in the metropolitan areas surrounding the large Spanish cities (dormitory cities from which the population commutes to work in the urban centers) (Fig. 4).

**Figure 5:**
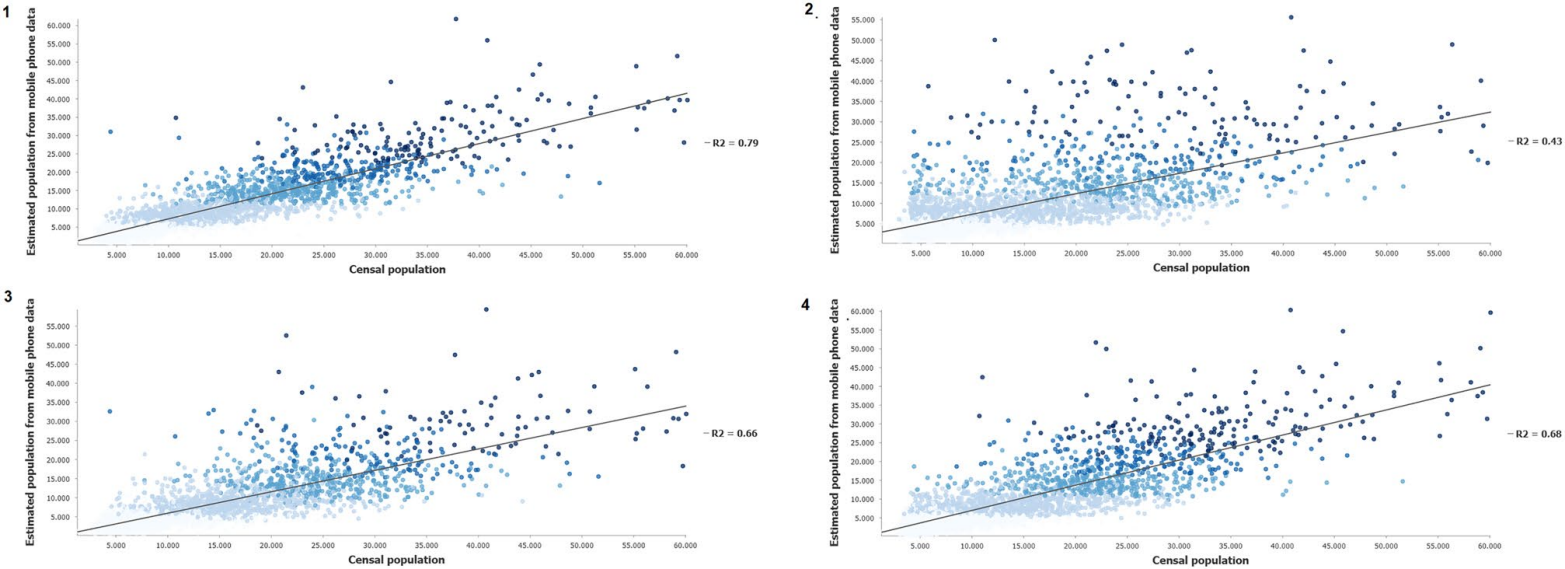
Coefficient of determination R^2^ between population from the 2020 census and population estimated from mobile phones on April 15, 2020 (1), August 19, 2020 (2), December 16, 2020 (3), and June 9, 2021 (4). *Source*: Own elaboration based on INE phone data from the *Estadistica Experimental* portal. The program used to create the figure is *ArcGIS Pro 3.0.3* GIS software. The figure is a dispersion diagram graph that shows the relationship of the estimated population values from mobile phone data and the number of inhabitants according to 2020 census data for the four mentioned days. The figure also indicates the R^2^ coefficients of determination for each diagram.

Determination coefficients have been used to visualize the adjustment of the population estimated from mobile phones with the INE population. During the day chosen to represent the first alarm state, an R^2^ value of 0.79 is observed, which confirms that there was a strong decrease in mobility due to confinement at the state level. In contrast, during the second state of alarm, the R^2^ value was 0.66, indicating higher levels of mobility due to the difference in rigidity or flexibility between the different autonomous communities in the establishment of mobility restriction measures. During the new normality period after the second state of alarm, the R^2^ value was 0.68, showing a good correlation too. These values contrast with that obtained in the 2020 holiday period, an R^2^ value of 0.43, which could mean a very high mobility in that period of the year (Fig. 5).

**Figure 6:**
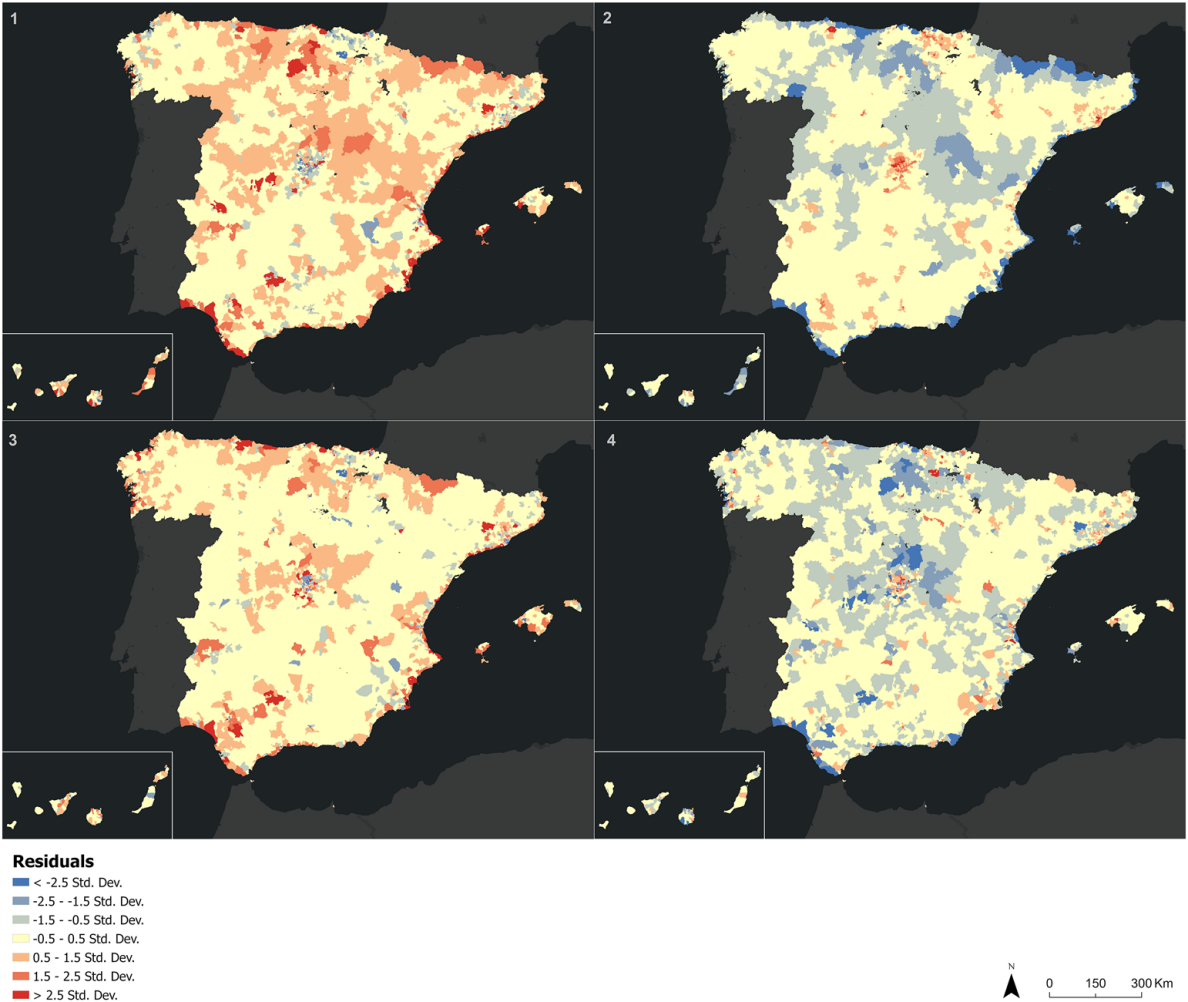
Spatial distribution of the calculated residuals between the INE population and the population estimated from cellmobile phones on April 15, 2020 (1), August 19, 2020 (2), December 16, 2020 (3), and June 9, 2021 (4). Source: Own elaboration based on INE phone data from the *Estadistica Experimental* portal. The program used to create the figure is *ArcGIS Pro 3.0.3* GIS software. The figure is a choropleth map that shows the residual values (overestimation or underestimation of estimated population) in a population cell for the four mentioned days. For that, an OLS analysis was performed using the estimated population from mobile phone data as the dependent variable and the 2020 censal data as the explanatory variable.

During the mobility restriction phases, the spatial distribution of the residuals calculated from an OLS analysis shows a strong overestimation of the population estimated from mobile phones (blue values) in the centers of the main cities of Spain, and an underestimation of this population (red values) in the adjacent dormitory cities and in the interior of the peninsula. This underestimation of the population is more visible during the first state of alarm. Meanwhile, the second state of alarm shows a generalized null standard deviation, while the underestimation of the estimated population is more concentrated on the Atlantic coast. On the opposite, there is an overestimation of the estimated population in most of the Spanish territory in the periods corresponding to the end of the mobility restrictions. In the 2020 summer period it’s noticeable how the main metropolitan areas have red values, indicating that their inhabitants left the major cities to travel to the second residences. The overestimation of the estimated population is more pronounced in the new normality period in 2021, coinciding with the normalization and progressive increase in mobility and activities of daily life (Fig. 6).

The Moran I spatial autocorrelation analysis shows that in all cases, the distribution of the residuals is clustered and spatially correlated. The values are very similar in three of the four cases, being the new normality period after the second state of alarm the striking situation due to its stronger values of clusterization (Table 1).

**Table 1:** Moran I results obtained from the calculated residuals between the INE population and the population estimated from mobile phones (*Source*: Own elaboration based on INE phone data).

Temporal period	Moran I value	Z value
April 15, 2020	0.133578	24.512012
August 19, 2020	0.307943	56.504751
December 16, 2020	0.094231	17.319788
June 9, 2021	0.127523	23.417660

## Conclusions

In order to analyze the special characteristics of the demography of Spain during the COVID-19 pandemic, it is necessary to have large volumes of data that can be easily updated and that allow monitoring of the population in high detail both spatially and temporally. This investigation has used CDR records from mobile phone data to map mobility and demographic patterns in the country during four different phases of the health crisis, with the goal to observe the usefulness of geolocated Big Data to extract and map behaviors associated to each phase and differences between them overall.

The results obtained adjust quite well with the findings from previous investigations. They show a strong decrease in mobility during states of alarm except in commuter cities associated with metropolitan areas^26^, showcasing that the trips were made mainly to local equipments^27^. Findings also include migration processes to second residence zones^25,34^, and a recovery of mobility as the weeks went by and the restrictions eased^30,32^. This consistency with respect to previous work shows the solidity of the findings of this research.

While previous investigations focused mainly on the first months of the COVID-19 pandemic, this work seeked to expand the temporal scope by analyzing four days corresponding to separate and established periods of the crisis, including both states of alarm Spain had in spring and fall of the year 2020, and the recovery to a normality situation corresponding to the supply of vaccines that was made to the Spanish population throughout the year 2021.

Thus, the insight from generated mobile phone data narrates how each of the four phases observed during the COVID-19 pandemic in Spain presents clear differentiations and its own characteristics. The first phase, associated with the rise of the virus and the first state of alarm, with strong confinement measures of the population, has a strong and obvious decrease in mobility in Spain (except in the main metropolitan areas for travels to basic infrastructures such as hospitals or pharmacies). In addition, there has been a general decline in the population throughout the country. The second study period, characterized by a lifting of mobility restriction measures, shows an increase in mobility flows to the coasts, archipelagos, and areas of second residence in the interior of the peninsula, creating a stronger clusterization in comparison with the other three periods of study. However, a more pronounced decrease in the population has also been seen, and this phenomenon can be linked to the high mortality suffered by Spain in the previous phase of the pandemic.

The third period, whose main characteristic consists of a different application of mobility restriction measures at the regional level, entails a new decrease in mobility, but to a lesser degree than with respect to the first state of alarm (although the patterns are similar, there is greater number of trips and travelers). In addition, it is in this period that a gradual recovery in population growth begins to be seen (although it is still negative, especially in the north and interior of the country). Finally, the last phase of the study, based on the vaccination campaign and the end of mobility restrictions, has a recovery in the number of trips in the territory, and a change in the growth trend of the country's population, which is beginning to be positive in various areas of the interior of the peninsula.

Overall, these findings have generated insight that fits very well which the phenomena observed in reality, indicating a strong consistency. In addition, the adjustment of the censal population with the estimated population obtained from mobile phone data has presented high values of coefficients of determination, especially during the alarm phases of the pandemic. This adjustment value is low, however, in the summer of 2020, which can be explained by traveling abroad or by the fact that mobile phone masts do not capture their users so well in inland areas associated with second homes. Therefore, in general terms it can be concluded that mobile phone data are a valid and reliable source of data for monitoring and analyzing the continuous changes in demographic and mobility patterns that occur during the different phases of a health crisis.

It should be noted that the use of mobile phone data comes with a few disadvantages. The spatial resolution of this data does not depend on the location of the user’s phone, but rather on the mobile phone antennas that pick up the calls. In areas where there is a large concentration of antennas such as large cities, the spatial resolution of the data is great, but, however, in areas where the antennas are in a dispersed manner such as in inland areas, the location of the phones leads to considerable errors. To alleviate this bias, INE has devised the population cells used in this study. It should also be kept in mind the privacy issues involved in using these types of data sources and the need to aggregate individual mobile phone records to try to mitigate these issues.

This work has several future lines of research planned such as the comparison of results other data sources such as Twitter, the study of demographic and mobility patterns in weekend periods with respect to weekdays, the enrichment of phone data with other data sources with socioeconomic information from each population cell, and the comparison of the results obtained with data from periods before or after the COVID-19 pandemic.

## Data Availability

Mobile phone data used for this work is openly available in the *Estadistica Experimental* portal by Instituto Nacional de Estadística in the data download section of the following url: https://www.ine.es/experimental/movilidad/experimental_em4.htm
